# Effective density of inhaled environmental and engineered nanoparticles and its impact on the lung deposition and dosimetry

**DOI:** 10.1186/s12989-024-00567-9

**Published:** 2024-02-17

**Authors:** Denisa Lizonova, Amogh Nagarkar, Philip Demokritou, Georgios A. Kelesidis

**Affiliations:** 1 Nanoscience and Advanced Materials Center (NAMC), Environmental and Occupational Health Science Institute, School of Public Health, Rutgers, The State University of New Jersey, 170 Frelinghuysen Road, Piscataway, NJ 08854 USA; 2 Particle Technology Laboratory, Department of Mechanical and Process Engineering, Institute of Process Engineering, ETH Zürich, Sonneggstrasse 3, 8092 Zurich, Switzerland

**Keywords:** Inhalation, Pulmonary deposition, Engineered nanoparticles, Air pollution, Black carbon, Wood smoke, Effective density

## Abstract

**Background:**

Airborne environmental and engineered nanoparticles (NPs) are inhaled and deposited in the respiratory system. The inhaled dose of such NPs and their deposition location in the lung determines their impact on health. When calculating NP deposition using particle inhalation models, a common approach is to use the bulk material density, *ρ*_*b*_, rather than the effective density, *ρ*_*eff*_. This neglects though the porous agglomerate structure of NPs and may result in a significant error of their lung-deposited dose and location.

**Results:**

Here, the deposition of various environmental NPs (aircraft and diesel black carbon, wood smoke) and engineered NPs (silica, zirconia) in the respiratory system of humans and mice is calculated using the Multiple-Path Particle Dosimetry model accounting for their realistic structure and effective density. This is done by measuring the NP *ρ*_*eff*_ which was found to be up to one order of magnitude smaller than *ρ*_*b*_. Accounting for the realistic *ρ*_*eff*_ of NPs reduces their deposited mass in the pulmonary region of the respiratory system up to a factor of two in both human and mouse models. Neglecting the *ρ*_*eff*_ of NPs does not alter significantly the distribution of the deposited mass fractions in the human or mouse respiratory tract that are obtained by normalizing the mass deposited at the head, tracheobronchial and pulmonary regions by the total deposited mass. Finally, the total deposited mass fraction derived this way is in excellent agreement with those measured in human studies for diesel black carbon.

**Conclusions:**

The doses of inhaled NPs are overestimated by inhalation particle deposition models when the *ρ*_*b*_ is used instead of the real-world effective density which can vary significantly due to the porous agglomerate structure of NPs. So the use of realistic *ρ*_*eff*_, which can be measured as described here, is essential to determine the lung deposition and dosimetry of inhaled NPs and their impact on public health.

**Graphical abstract:**

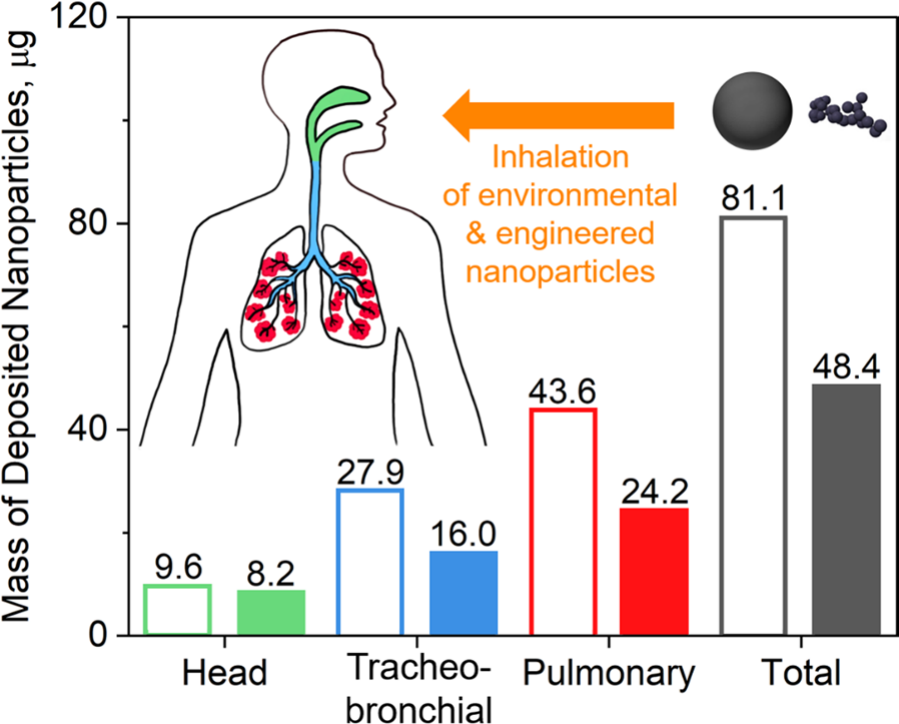

**Supplementary Information:**

The online version contains supplementary material available at 10.1186/s12989-024-00567-9.

## Background

Over the past century, the exposure of humans to airborne environmental and engineered nanoparticles (NPs) has increased dramatically due to air pollution, technological advancements and use in nano-enabled products across the value chain and various industries [1–6]. Such nanoscale particles share unique physicochemical properties that stem from their small size and large surface area, chemistry and reactivity and render them rather toxic to human health [7].

In particular, environmental and engineered NPs have been linked with a variety of pulmonary [8–11], cardiovascular [12–15] and other effects [16–19], even though the underlying mechanisms are still not well understood. It is worth noting that given the continuous rise of air pollution due to climate change [20, 21], as well as the emerging markets for engineered nanomaterials [22], it is essential to get a better understanding of the impact of these NPs on public health.

Most of the (primary) airborne environmental pollutants, such as black carbon (BC) or wood smoke, are emitted from combustion sources, including engines, coal or biomass combustors and wildfires [23–25]. In addition to environmental pollutants, combustion contributes decisively to the formation of nanostructured commodities, including carbon black, silica and titania that are produced in flame reactors [22]. The environmental and engineered NPs formed during these processes coagulate into porous, fractal-like clusters (i.e. agglomerates) [26, 27]. The size of these agglomerates is commonly quantified by their mobility and aerodynamic diameters [28] that vary significantly between materials and combustion sources and processes, as summarized in Table 1. The agglomerate porosity is determined by the effective density, *ρ*_*eff*_, that is defined here as the ratio of the particle mass and equivalent mobility volume and is just a fraction of the material bulk density, *ρ*_*b*_ [28, 29]. The small agglomerate *ρ*_*eff*_ affects the gravitational settling, inertial impaction and diffusion of NPs [30] and thus affects their lung deposition and dosimetry.

**Table 1:** Count Median (CMD), Mass Median Mobility (MMMD), Mass Median Aerodynamic (MMAD) diameters, median *ρ*_*eff*_ and bulk density, *ρ*_*b*_, used in the Multiple-Path Particle Dosimetry (MPPD) model for the estimation of deposited NP mass

	Aircraft BC	Diesel BC [65]	Wood Smoke [64]	Silica [70]	Zirconia [71]
CMD, nm	107.8	88.0	159.1	97.3	68.1
MMMD, nm	182.9	349.5	309.5	182.2	132.2
MMAD, nm	83.2	152.8	142.9	65.1	98.8
*ρ*_*eff*_, g/cm^3^	0.34	0.28	0.31	0.26	0.68
*ρ*_*b*_, g/cm^3^	1.8	1.8	1.7	2.2	5.7

In nanotoxicology research, both in vivo animal studies as well as in vitro cellular approaches are employed to assess potential toxicological endpoints [31, 32]. Particle lung deposition models such as the Multiple-Path Particle Dosimetry (MPPD) [33, 34] and International Commission on Radiological Protection (ICRP) [35] models, are often used to determine the lung deposited dose using the airborne exposure levels of inhaled NPs. For example, MPPD has been recently used by the authors and others to derive the inhaled dose of ambient particulate matter [36–38], BC [39, 40], wood smoke [41], titania [42], ceria [31, 43], micro- and nanoplastics [44], nano-enabled products [45], printer emitted particles [46, 47] and e-cigarette [48] emissions using *ρ*_*b*_ rather than *ρ*_*eff*_. From the calculated in vivo lung-deposited dose, the in vitro administered dose can also be back-calculated using in vitro particle-kinetic dosimetry models, as described in detail by the authors in previous publications [3, 31, 45, 49, 50]. It should be noted that the effective density for in vitro particle dosimetry is defined as the density of the formed agglomerate in a culture medium [49, 50].

For simplicity, MPPD is commonly employed using *ρ*_*b*_ which can differ significantly from the *ρ*_*eff*_*.* [31, 38, 39]. This oversimplification may limit though the accuracy of MPPD calculations for various environmental and engineered NPs that form agglomerates with small *ρ*_*eff*_ [26]. For example, the total deposited mass of ceria NPs measured in mice was overestimated by MPPD using the ceria *ρ*_*b*_ by up to a factor of two [43]. Similarly, the mass of diesel BC deposited in the human respiratory system obtained using *ρ*_*b*_ (1 g/cm^3^) was a factor of two larger than that derived using the measured *ρ*_*eff*_ [51].

In this regard, the development and commercialization of aerosol particle mass (APM) analyzers have enabled the accurate measurement of the NP *ρ*_*eff*_ [52–55]. During APM measurements, NPs pass through an electric field between two rotating cylindrical electrodes. By adjusting the electric field potential and the rotating electrode angular velocity, the particle mass [52], volume fraction [56] and consequently *ρ*_*eff*_ [57] can be measured. It should be noted that APM is well suited for characterization of NP agglomerates, but its accuracy is not well established for elongated particles (e.g. fibers or tubes). For example, the alignment of such particles in an external electric field [58] can result in measurement errors up to 7% [59]. In addition to the APM analyzers, *ρ*_*eff*_ can be also measured using electrical low pressure [60] or hypersonic impactors [61] and time-of-flight mass spectrometers [62]. The agglomerate *ρ*_*eff*_ can be obtained also in vivo by fitting the MPPD simulations to the measured lung burden [63].

So, APM analyzers have been used to obtain the *ρ*_*eff*_ of environmental NPs, including wood smoke [64], BC emissions from diesel [57, 65], gasoline [66, 67] and marine [68] engines, as well as that of engineered nanomaterials (e.g. carbon black [69], silica [70], zirconia [71]). The *ρ*_*eff*_ measured that way has facilitated the derivation and validation of advanced computational models [72] for the particle morphology [26], light absorption [73, 74], scattering [75, 76] and even climate impact [77].

Here, APM is used to demonstrate how to measure the *ρ*_*eff*_ of model environmental NPs, namely, aircraft-like BC from enclosed jet fuel combustion [78]. The aircraft BC *ρ*_*eff*_ obtained here, as well as those of other model NPs obtained from the literature for diesel BC [65], wood smoke [64], silica [70] and zirconia [71] (summarized in Table 1) are used in MPPD to determine the error from dose calculations derived using the commonly used pristine material bulk density. The deposited mass distributions derived using *ρ*_*eff*_ are validated with experimental data of human exposure diesel BC emissions [65] and compared to those obtained commonly in the literature using *ρ*_*b*_.

## Results and discussion

### Effective density of environmental and engineered NPs

**Fig. 1:**
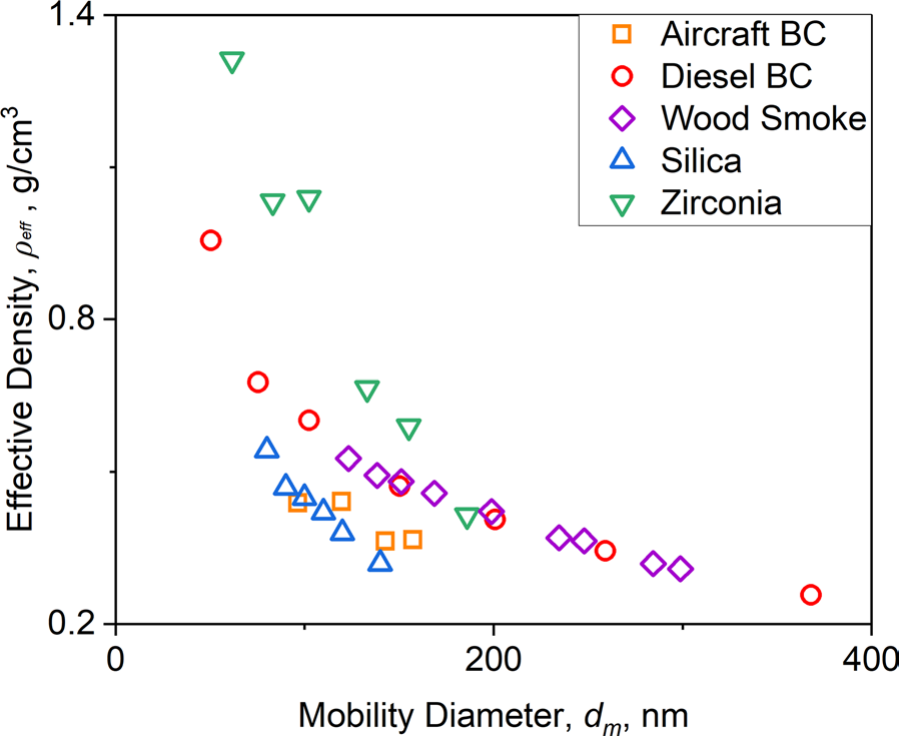
Effective density, *ρ*_*eff*_*,* as a function of the mobility diameter, *d*_*m*_, measured for aircraft (squares) or diesel BC (circles [65]), wood smoke (diamonds [64]), silica (triangles [70]) and zirconia (inverse triangles [71]) NPs

Figure 1 shows the *ρ*_*eff*_ measured for various model NPs such as aircraft (squares, this work) or diesel BC (circles [65]), wood smoke (diamonds [64]), silica (triangles [70]) and zirconia (inverse triangles [71]) as a function of their mobility diameter, *d*_*m*_. The raw *ρ*_*eff*_ data presented in Fig. 1 have been obtained for NP agglomerates with distinct *d*_*m*_. The NP *ρ*_*eff*_ decreases up to a factor of about four with increasing *d*_*m*_ due to their fractal-like, agglomerate morphology, which is consistent with theoretical [26] and empirical [79] power laws derived for agglomerates. The *ρ*_*eff*_ of zirconia NPs is up to factor of two larger than those of BC, wood smoke and silica due to their larger *ρ*_*b*_ (see Table 1). Similarly, the *ρ*_*eff*_ measured here for aircraft BC is up to a factor of 1.4 smaller than that of diesel BC and wood smoke NP agglomerates having the same *d*_*m*_. The bulk density, *ρ*_*b*_, is practically the same for aircraft, diesel BC and wood smoke primary particles (Table 1). So, this *ρ*_*eff*_ difference can be attributed to the diameter of about 28 nm of diesel BC [65] and wood smoke [64] primary particles that is 50% larger than the diameter of aircraft BC primary particles (12 nm [78]). This is consistent with theoretical power laws showing that *ρ*_*eff*_ increases with the primary particle diameter [26]. It is worth noting that the *ρ*_*eff*_ presented here for environmental and engineered NPs is up to an order of magnitude smaller than the respective *ρ*_*b*_.

Using the measured *ρ*_*eff*_ along with the entire mobility size distribution, one can obtain the overall NP mass median mobility diameter (MMMD), as well as the mass median aerodynamic diameter (MMAD; see Methods). The latter is essential for the estimation of the NP lung deposition and dose. Even though zirconia NPs have larger *ρ*_*eff*_ compared to silica (Fig. 1), their *d*_*m*_ obtained from the entire size distribution is about 30% smaller. This explains the MMMD of silica NPs that is 27% larger than that of zirconia ones. Table 1 summarizes the count median diameter (CMD), MMMD, MMAD, *ρ*_*b*_ and *ρ*_*eff*_ of agglomerates having MMAD and MMMD for all NPs used in this study. For example, diesel BC agglomerates with MMAD = 152.8 nm and MMMD = 349.5 nm have *ρ*_*eff*_ = 0.28 g/cm^3^, which is within the *ρ*_*eff*_ = 0.96–0.26 g/cm^3^ measured for agglomerates with *d*_*m*_ = 50–368 nm (Fig. 1: circles [65]).

### Lung deposition calculations and validation of MPPD dosimetric calculations with human experimental data using *ρ*_*eff*_

**Fig. 2:**
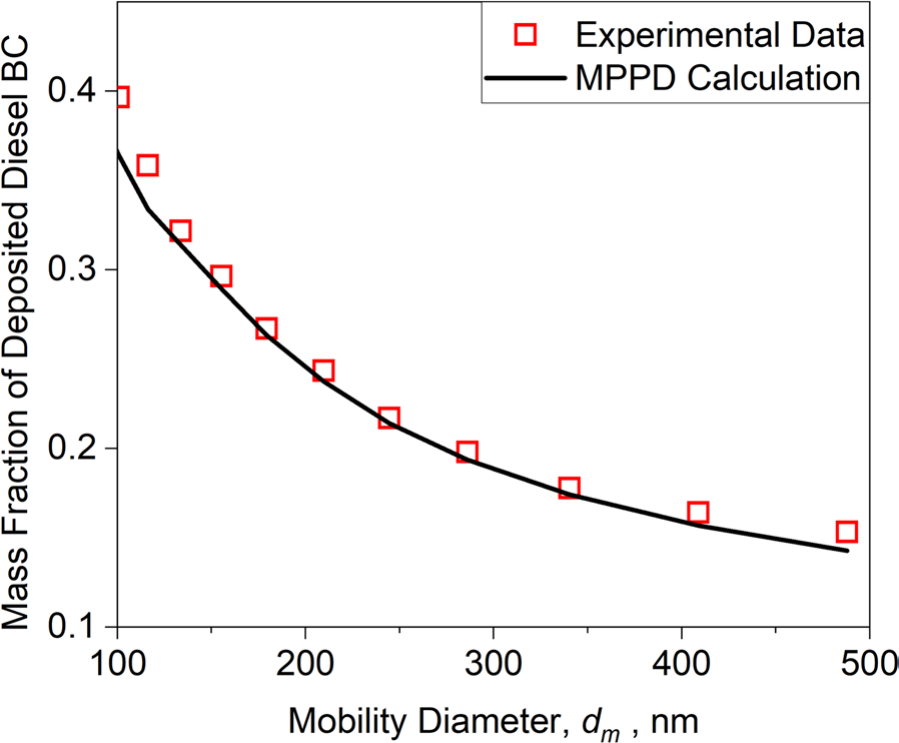
Mass fraction of deposited diesel BC NPs measured (symbols [65]) or estimated by MPPD (line) as a function of *d*_*m*_

Lung deposition of inhaled NPs was simulated using MPPD with realistic *ρ*_*eff*_ (Fig. 2) and validated with measurements for the case of diesel BC [65]. The deposited mass fractions derived here by MPPD accounting for the realistic *ρ*_*eff*_ of diesel BC are in excellent agreement with the measured ones, validating the MPPD simulations presented in this work. In particular, Fig. 2 compares the mass fraction of deposited diesel BC as a function of its *d*_*m*_ derived by MPPD using *ρ*_*eff*_ (line) to those measured from 9 human subjects exposed to the exhaust of a real diesel engine (symbols [65]). These data were obtained using the *d*_*m*_ distributions measured in the inhaled and exhaled air. The *ρ*_*eff*_ used in MPPD is varied with *d*_*m*_ using Eq. 2 (see Methods) with mass-mobility exponent and prefactor derived by fitting Eq. 2 to the *ρ*_*eff*_ measured for diesel BC (see Additional file 1: Table S1). At this size range, the deposition of diesel BC particles by diffusion, inertial impaction and gravitational settling decreases with increasing *d*_*m*_ [80, 81], reducing the total deposited mass fraction.

### Impact of* ρ*_*b*_ and *ρ*_*eff*_ on lung deposition dose calculations

**Fig. 3:**
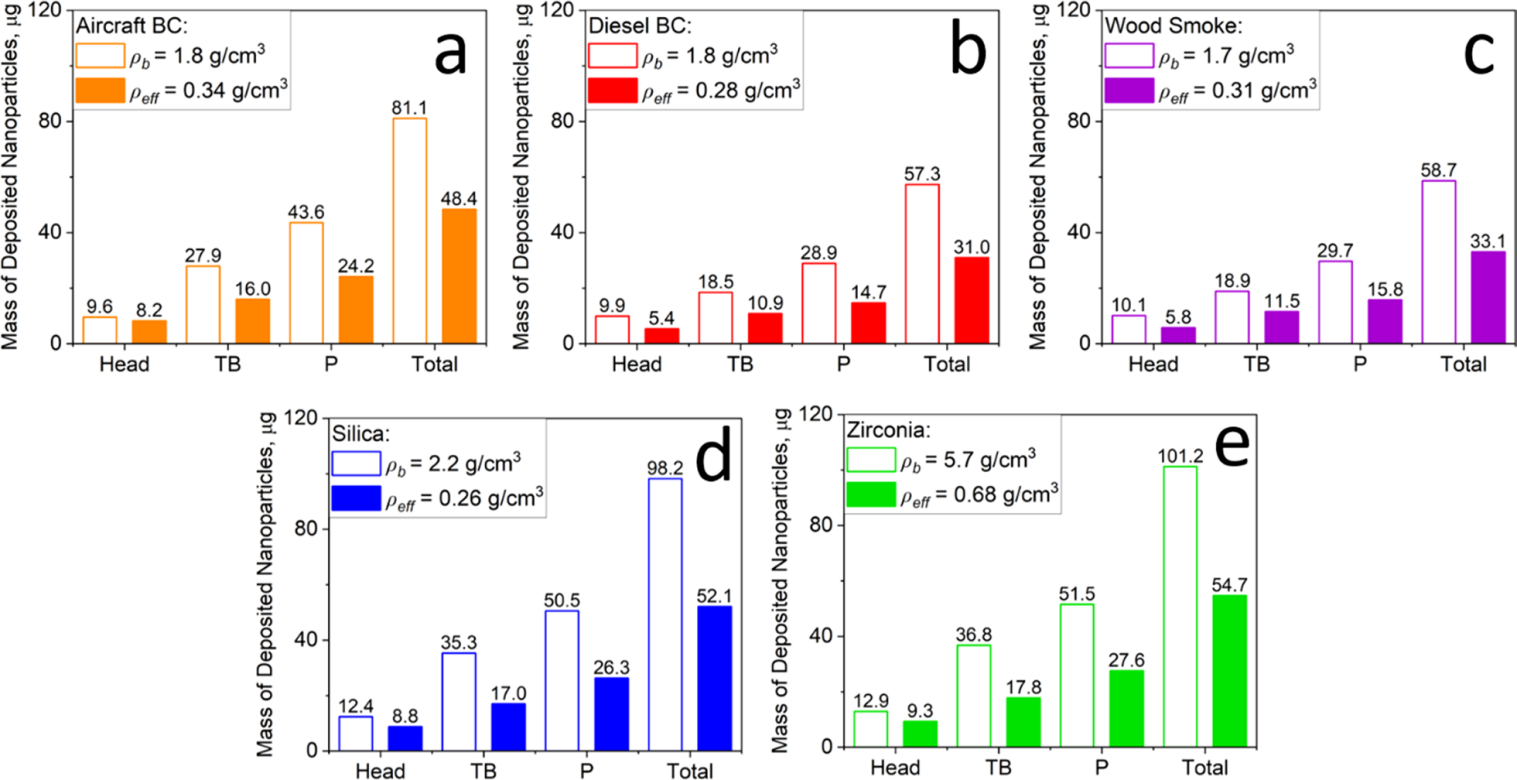
Mass of deposited NPs in the head, tracheobronchial (TB), pulmonary (P) and total region of the human respiratory tract derived by MPPD for a 40-h exposure to **a** aircraft, **b** diesel BC, **c** wood smoke, **d** silica or **e** zirconia NPs using *ρ*_*b*_ (open bars) or the measured *ρ*_*eff*_ (filled bars). The total inhaled dose is 180 µg

**Fig. 4:**
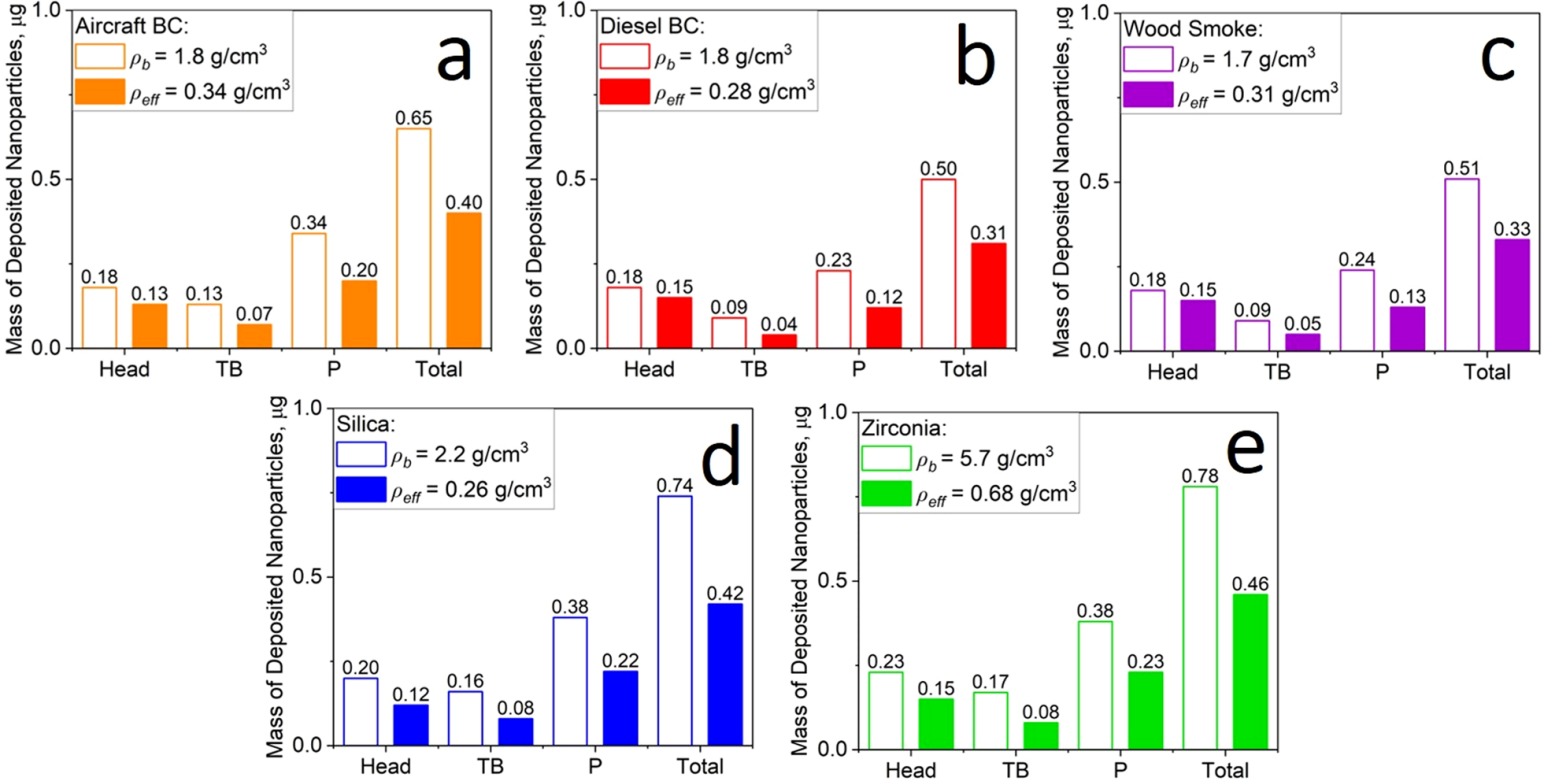
Mass of deposited NPs in the head, TB, P and total region of the mouse respiratory tract derived by MPPD for a 40-h exposure to **a** aircraft, **b** diesel BC, **c** wood smoke, **d** silica or **e** zirconia NPs using *ρ*_*b*_ (open bars) or the measured *ρ*_*eff*_ (filled bars). The total inhaled dose is 1.2 µg

The deposited mass of environmental and engineered NPs in the respiratory tract of humans (Fig. 3) and mice (Fig. 4) was calculated using the MPPD model with the measured *ρ*_*eff*_ (filled bars) or *ρ*_*b*_ (open bars) under the same input parameters. It is worth noting that MPPD, like any other model, has its own limitations and more studies are needed to validate the model for the various conditions and animal models. In humans (Fig. 3), using *ρ*_*b*_ rather than *ρ*_*eff*_ results in an overestimation of the total deposited mass by a factor of about two for all environmental and engineered NPs investigated here. Neglecting the realistic agglomerate *ρ*_*eff*_ affects also the regional distribution of the deposited mass. For example, using *ρ*_*b*_ in MPPD overestimates the deposited mass of aircraft BC in the head human airways by just 17.1%. However, the deposited aircraft BC mass in the tracheobronchial and pulmonary regions is overestimated using *ρ*_*b*_ by 74.4 and 80.2%, respectively.

The overestimation of the deposited mass of NPs can be attributed to the enhancement of the particle inertial impaction in the TB and pulmonary regions when large *ρ*_*b*_ is used instead of the realistic *ρ*_*eff*_ [30]. It should be noted that gravitational settling hardly contributes to the density effects observed here (Additional file 1: Fig. S1). So, the *ρ*_*b*_ is commonly assumed in literature estimations of inhaled NP deposition and dosimetry when the realistic *ρ*_*eff*_ is either not known, or for the purpose of simplifying the calculations [38, 39, 43]. This can however lead to significant error in the NP deposition calculation, as is shown here.

Figure 3 shows that most of the particles are deposited in the tracheobronchial (TB) and pulmonary (P) regions of the human respiratory system, where the deposition is governed by diffusion, inertial impaction and gravitational settling [80–82]. Therefore, the largest deposited mass was obtained for zirconia NPs. These NPs are described by small MMAD compared to those of diesel BC and wood smoke which enhances their deposition by diffusion [80, 82]. Moreover, zirconia NPs are described by high *ρ*_*b*_ and *ρ*_*eff*_ compared to those of aircraft BC and silica, which further enhance their inertial impaction [30]. In the head airways of the human respiratory system, only few particles are deposited in all cases, where this is done by an impaction mechanism [80, 81, 83].

The impact of *ρ*_*eff*_ on the estimation of the NP deposited dose is similar for both human and mouse models, as shown in Fig. 4. So, neglecting the realistic *ρ*_*eff*_ and calculating with *ρ*_*b*_ instead results in an overestimation of the total NP deposited mass in mouse lungs by up to a factor of about two. The largest mass deposited in the TB and pulmonary regions is obtained here for zirconia, consistent with the masses derived for zirconia NPs inhaled by humans (Fig. 3).

In summary, Table 2 shows the total mass of deposited NPs in the human and mouse respiratory tracts derived by MPPD using *ρ*_*b*_ or *ρ*_*eff*_. The overestimation of the total deposited mass by a factor of 1.5–2 obtained here using MPPD with *ρ*_*b*_ is consistent with those reported in literature for engineered [43] and environmental [51] NPs. Clearly, the dose of inhaled engineered and environmental NPs can be overestimated substantially by MPPD using *ρ*_*b*_, limiting the assessment of their impact on pulmonary [8, 9] and cardiovascular diseases [12–15].

**Table 2:** Total mass of deposited aircraft, diesel BC, wood smoke, silica and zirconia NPs in the human and mouse respiratory tracts derived by MPPD using *ρ*_*b*_ or *ρ*_*eff*_. The total inhaled dose is 180 and 1.2 µg for human and mouse, respectively

Total mass of deposited NPs, μg	Aircraft BC		Diesel BC		Wood smoke		Silica		Zirconia	
	Human	Mouse	Human	Mouse	Human	Mouse	Human	Mouse	Human	Mouse
MPPD using *ρ*_*b*_	81.1	0.65	57.3	0.50	58.7	0.51	98.2	0.74	101.2	0.78
MPPD using *ρ*_*eff*_	48.4	0.40	31.0	0.31	33.1	0.33	52.1	0.42	54.7	0.46

**Fig. 5:**
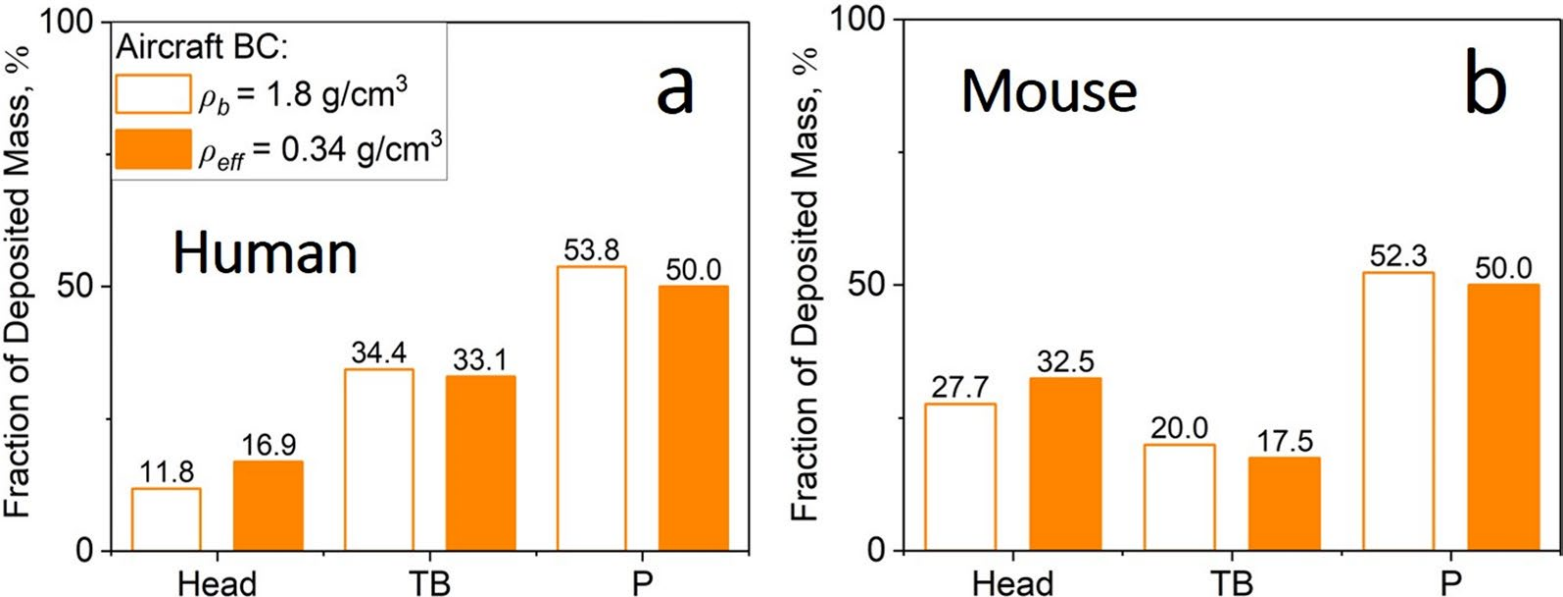
Fraction of deposited mass in the head, TB and P region of the **a** human and **b** mouse respiratory tract derived by MPPD for a 40-h exposure to aircraft BC using *ρ*_*b*_ (open bars) or the measured *ρ*_*eff*_ (filled bars)

Furthermore, Fig. 5 shows the distribution across the respiratory system of the deposited mass fraction of inhaled aircraft BC NPs by humans (a) and mice (b) derived here by MPPD using *ρ*_*b*_ (open bars) or *ρ*_*eff*_ (filled bars). The deposited mass fraction is obtained by normalizing the mass deposited in the head, TB or pulmonary region of the tract with respect to the total deposited mass. Accounting for the realistic *ρ*_*eff*_ of aircraft BC reduces its inertial impaction in all regions of the human or mouse respiratory tract and does not alter significantly the distribution of the deposited mass fractions. The distributions of the deposited mass fractions derived here for diesel BC, wood smoke, silica and zirconia are similar to those obtained for aircraft BC and presented in Additional file 1: Fig. S2.

## Conclusions

In sum, the error in NP lung deposition dose calculations which is derived using the *ρ*_*b*_ rather than the actual *ρ*_*eff*_ of NPs was assessed here using a variety of model environmental and engineered NPs. As shown, the *ρ*_*eff*_ measured here for aircraft black carbon (BC) NPs using an APM is one order of magnitude smaller than *ρ*_*b*_ and follows closely those measured in literature for diesel BC [65], wood smoke [64], silica [70] and zirconia [71]. It was shown that the MPPD-derived mass fraction of diesel BC NPs deposited in the human lungs is in excellent agreement with experimental data [65], validating the importance of using the realistic *ρ*_*eff*_ rather than the commonly used *ρ*_*b*_.

More importantly, it was shown that using *ρ*_*b*_ and neglecting the realistic porous structure of environmental and engineered NPs results in an overestimation of their deposited mass by a factor of about two. This can be attributed to the NP inertial impaction that is overestimated by MPPD using *ρ*_*b*_ instead of *ρ*_*eff*_. This may explain similar discrepancies reported in literature for ceria [43] and diesel BC [51] NPs and highlights the role of *ρ*_*eff*_ in the modeling of lung deposition of NPs. So, the use of realistic *ρ*_*eff*_ in lung deposition models is essential to determine the dose of inhaled NPs, enabling the accurate assessment of their impact on human health.

## Methods

### Synthesis of aircraft-like BC NPs and measurement of their size and effective density

Aircraft-like BC NPs were generated here by enclosed spray combustion of jet A fuel at an effective equivalence ratio of 1.77 [78]. The morphology, composition, nanostructure and primary particle size distribution of the BC NPs emitted by the present reactor (Additional file 1: Fig. S3) are in excellent agreement with those measured from real aircraft engines [84, 85]. So, the aircraft-like BC produced here was sampled using a straight tube and rapidly diluted by a factor of about 65 [71]. The diluted aerosol was directed to a scanning mobility particle sizer (SMPS) made of a differential mobility analyzer (Model 3081, TSI Inc.) coupled with a condensation particle counter (Model 3775, TSI Inc.) [71]. The CMD and MMMD of the *d*_*m*_ distribution obtained by SMPS are given in Table 1. The mass, *m*, of the sampled aerosol was also measured by interfacing an aerosol particle mass (APM, Model APM-3600, Kanomax) analyzer with the SMPS [86]. That way, the *ρ*_*eff*_ can be derived from first principles [28]:

1 $$\rho_{eff} = \frac{m}{{\frac{\pi }{6}d_{m}^{3} }}$$

The NP agglomerate *ρ*_*eff*_ measured this way decreases with *d*_*m*_ based on a power law [87]:

2 $$\rho_{eff} = \frac{6k}{\pi }d_{m}^{D_{fm} - 3}$$

where *k* and *D*_*fm*_ are the mass-mobility prefactor and exponent, respectively. The NP agglomerate *k* and *D*_*fm*_ were derived by fitting Eq. 2 to the data shown in Fig. 1 (Additional file 1: Table S1). So, *ρ*_*eff*_ can be estimated for any *d*_*m*_ using Eq. 2 and the fitted *k* and *D*_*fm*_. MMAD was derived based on the measured MMMD and *ρ*_*eff*_ [87, 88]:

3 $${\text{MMAD}} = {\text{MMMD}} \sqrt {\frac{{\rho_{eff} C_{C} \left( {{\text{MMMD}}} \right)}}{{\rho_{o} C_{C} \left( {{\text{MMAD}}} \right)}}}$$

where $\rho_{o}$ = 1 g/cm^3^ is the unitary density and *C*_*C*_ is the Cunningham slip correction factor [80]:

4 $$C_{C} \left( d \right) = 1 + \frac{2\lambda }{d}\left( {1.257 + 0.4\exp ( - 0.78d/\lambda } \right)$$

where *d* = MMMD or MMAD and *λ* = 66 nm is the gas mean free path at room temperature [80]. The MMAD was obtained for aircraft BC NPs generated here, as well as for the diesel BC [65], wood smoke [64], silica [70] and zirconia [71] NPs using *ρ*_*eff*_ and *d*_*m*_ distribution data available in the literature (Table 1).

### Simulation of NP deposition in the respiratory system using MPPD model

The MPPD model (V3.04) was used here to simulate the deposition of inhaled engineered and environmental NPs in the lung airway from the head to the alveolar region [33, 34, 89, 90]. MPPD calculations for humans were done using the Yeh/Schum symmetric model [91] with a functional residual capacity of 3300 mL and head volume of 50 mL [92]. The nasal respiratory rate (RR) was set to 12 breaths/minute, the tidal volume (TV) to 625 mL and the inspiratory fraction to 0.5 [92]. MPPD calculations were also done for mice using the mouse BALB/c model [33] with body weight of 30 g [93]. The RR of 224 breaths/min and TV of 0.22 mL derived for mice using the allometric scaling equations of Guyton et al. [94] and Piccione et al. [95], respectively, were used for input into MPPD. The functional residual capacity (FRC) of 0.3 mL was used to be consistent with the measured range of 0.20–0.43 mL [96]. The upper respiratory tract (URT) volume of 0.0322 mL used here is the default MPPD value, which is based on experimental measurements [93] and is commonly utilized in MPPD simulations [97, 98]. Both humans and mice were assumed to be exposed to a particle concentration of 0.01 mg/m^3^ at “upright” and “on stomach” body orientations, respectively. The latter is consistent with in vivo conscious animal studies [99]. The mass concentration of 0.01 mg/m^3^ is the proposed PM_2.5_ limit by United States Environmental Protection Agency (EPA) [100]. It should be noted that PM_2.5_ contains larger particles than those investigated here that are largely contained in the PM_0.1_ aerodynamic size fraction. In this regard, the mass concentration of PM_0.1_ emissions from the combustion sources investigated here are often much larger than the EPA PM_2.5_ limit used here. For example, mass concentrations of 3.3–26, 0.6–0.8 and 0.004–0.5 mg/m^3^ have been measured from pinewood [24], diesel [101], and jet fuel [102] combustion, respectively. The MPPD parameters are summarized in Additional file 1: Table S2. The inhaled NPs were assumed to be monodisperse having the measured MMAD and *ρ*_*eff*_ (Table 1) or the constant bulk densities, *ρ*_*b*_ = 1.8, 1.7, 2.2 and 5.7 g/cm^3^ for BC, wood smoke, silica and zirconia, respectively. The wood smoke *ρ*_*b*_ is obtained based on the measured organic carbon content and empirical *ρ*_*b*_ relations [74]. The deposited mass is calculated from the MPPD-derived regional deposited mass rate per minute (µg/min) by integrating over 40 h of exposure (equivalent to 8 h per day, 5 days per week), as previously described by Bitounis et al. [10]. It is worth noting that MPPD, despite its wide use in the nanotoxicology domain, has its own limitations (like any other available inhalation dosimetry model) and further validation studies related to its proposed conditions and animal models will be useful in advancing the dosimetry field.

The impact of the *ρ*_*eff*_ variation with *d*_*m*_ on the MPPD calculations was also investigated here. To this end, the lung deposition of aircraft BC was simulated assuming monodisperse particles with MMAD, as well as accounting for their polydispersity by discretizing their *d*_*m*_ distribution into 10 bins (Additional file 1: Table S3) using Eq. 2 with *k*, *D*_*fm*_ derived by fitting Eq. 2 to the *ρ*_*eff*_ measured for aircraft BC (Additional file 1: Table S1). Accounting for the geometric standard deviation of the mobility size distribution, as well as for the *ρ*_*eff*_ variation with *d*_*m*_ decreased the total deposited mass just by 6% (Additional file 1: Fig. S4). Therefore, the lung deposition of inhaled NPs can be estimated rather accurately neglecting their polydispersity.

### Supplementary Information

**Additional file 1:** Supplementary Information including** Figure S1.** Mass of deposited NPs in the head, TB, P and total region of the human respiratory tract derived by MPPD for a 40-h exposure to aircraft black carbon using the bulk or the measured effective density and gravitational constants of 9.81 (**a**) or 0 m/s (**b**);** Figure S2.** Fraction of deposited mass in the head, TB and P region of the human and mouse respiratory tract derived by MPPD for a 40-h exposure to **a**-**b** diesel BC, **c**-**d** woodsmoke,** e**-**f** silica and** g**-**h** zirconia using the bulk or the measured effective density;** Figure S3.** Schematic of the experimental set up for preparation of aircraft black carbon nanoparticles from enclosed spray combustion of jet fuel;** Figure S4.** Mass of deposited aircraft black carbon nanoparticles in the human respiratory tract derived by MPPD for a 40-h exposure based on one bin or the mass-weighted average of 10 bins used to discretize the mobility size distribution of aircraft black carbon;** Table S1.** Summary of the massmobility prefactor and exponent derived by fitting Eq. 2 to the measured effective densities shown in Fig. 1;** Table S2.** Summary of parameters used for the particle deposition calculations for humans and mice using the MPPD model (V3.04);** Table S3.** Count Median Diameter, Mass Median Aerodynamic Diameter, bin median effective density and mass fraction of aircraft black carbon having a mobility size distribution discretized into 10 bins.

## Data Availability

All data generated or analyzed during this study are included in this published article and its supplementary information file.

## Abbreviations

APM: Aerosol particle mass analyzer
BC: Black carbon
CMD: Count median diameter
EPA: United States Environmental Protection Agency
ICRP: International Commission on Radiological Protection
MMAD: Mass median aerodynamic diameter
MMMD: Mass median mobility diameter
MPPD: Multiple-path particle dosimetry model
NPs: Nanoparticles
P: Pulmonary
PM: Particulate matter
SMPS: Scanning mobility particle sizer
TB: Tracheobronchial

## List of symbols

*C*_*C*_: Cunningham slip correction factor
*d*: Diameter (nm)
*d*_*m*_: Mobility diameter (nm)
*m*: Mass (kg)

## Greek letters

*λ*: Gas mean free path (nm)
*ρ*_*o*_: Unitary density (1 g/cm^3^)
*ρ*_*b*_: Bulk density (g/cm^3^)
*ρ*_*eff*_: Effective density (g/cm^3^)
