# Supplementary material for: Effective density of inhaled environmental and engineered nanoparticles and its impact on the lung deposition and dosimetry
Source: Part Fibre Toxicol. 2024 Feb 17;21:7. doi: 10.1186/s12989-024-00567-9 (PMC10874077; doi:10.1186/s12989-024-00567-9)
Supplement: Supplementary file 1 — Additional file 1: Supplementary Information including Figure S1. Mass of deposited NPs in the head, TB, P and total region of the human respiratory tract derived by MPPD for a 40-h exposure to aircraft black carbon using the bulk or the measured effective density and gravitational constants of 9.81 (a) or 0 m/s (b); Figure S2. Fraction of deposited mass in the head, TB and P region of the human and mouse respiratory tract derived by MPPD for a 40-h exposure to a-b diesel BC, c-d woodsmoke, e-f silica and g-h zirconia using the bulk or the measured effective density; Figure S3. Schematic of the experimental set up for preparation of aircraft black carbon nanoparticles from enclosed spray combustion of jet fuel; Figure S4. Mass of deposited aircraft black carbon nanoparticles in the human respiratory tract derived by MPPD for a 40-h exposure based on one bin or the mass-weighted average of 10 bins used to discretize the mobility size distribution of aircraft black carbon; Table S1. Summary of the massmobility prefactor and exponent derived by fitting Eq. 2 to the measured effective densities shown in Fig. 1; Table S2. Summary of parameters used for the particle deposition calculations for humans and mice using the MPPD model (V3.04); Table S3. Count Median Diameter, Mass Median Aerodynamic Diameter, bin median effective density and mass fraction of aircraft black carbon having a mobility size distribution discretized into 10 bins. [file 12989_2024_567_MOESM1_ESM.docx]

*Supplementary Information For*

**Effective density of inhaled environmental and engineered nanoparticles and its impact on the lung deposition and dosimetry**

*Denisa Lizonova^1^, Amogh Nagarkar^2^, Philip Demokritou^1^, Georgios A. Kelesidis^1,2*^*

^1^Nanoscience and Advanced Materials Center (NAMC), Environmental and Occupational Health Science Institute, School of Public Health, Rutgers, The State University of New Jersey, 170 Frelinghuysen Road, Piscataway, NJ, 08854, USA

^2^Particle Technology Laboratory, Institute of Process Engineering, Department of Mechanical and Process Engineering, ETH Zürich, Sonneggstrasse 3, CH-8092 Zürich, Switzerland

*Corresponding author: georgios.kelesidis@rutgers.edu

8 Pages

3 Tables

4 Figures**Summary**

Figure S1....................................................................................................................................p. S3

S2....................................................................................................................................p. S4

S3....................................................................................................................................p. S5

S4....................................................................................................................................p. S6

Table S1....................................................................................................................................p. S7

S2....................................................................................................................................p. S7

S3....................................................................................................................................p. S8


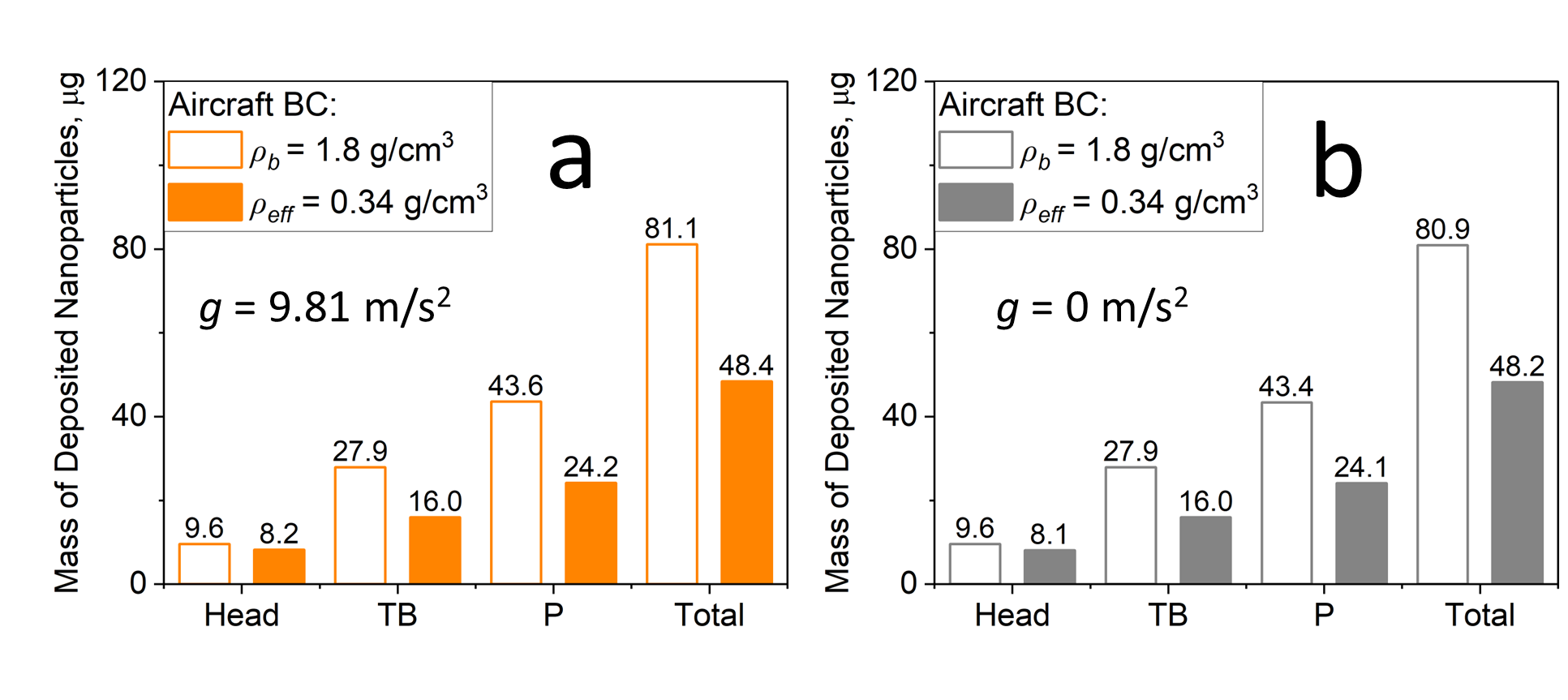


**Figure S1**. Mass of deposited NPs in the head, tracheobronchial (TB), pulmonary (P) and total region of the human respiratory tract derived by MPPD for a 40-hour exposure to aircraft BC using the bulk density, *ρ_b_* (open bars), or the measured effective density, *ρ_eff_* (filled bars), and gravitational constant, *g* = 9.81 (a) or 0 m/s^2^ (b). The total inhaled dose is 180 µg.


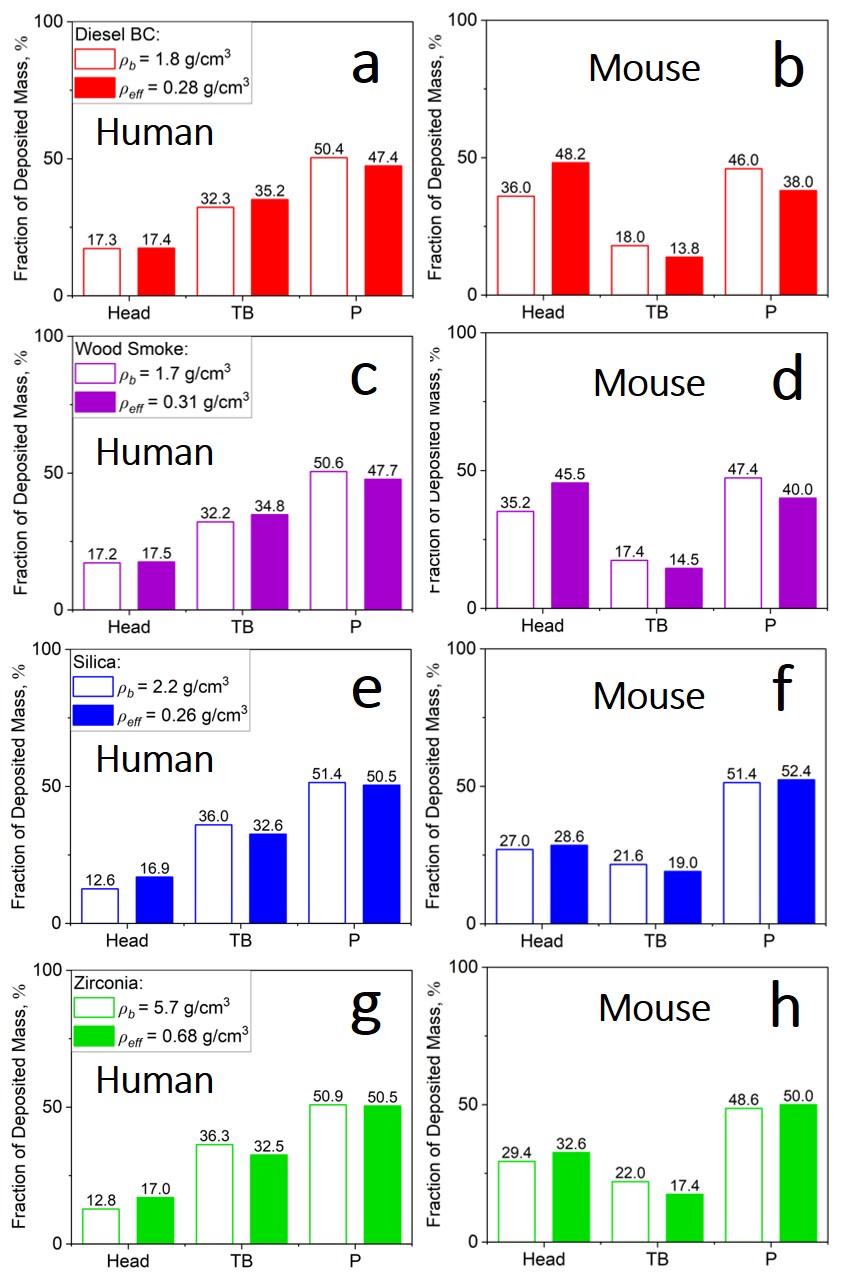


**Figure S2.** Fraction of deposited mass in the head, TB and P region of the human and mouse respiratory tract derived by MPPD for a 40-hour exposure to (a-b) diesel BC, (c-d) woodsmoke, (e-f) silica, and (g-h) zirconia using bulk density, ρ_b_ (open bars) or the measured effective density, ρ_eff_ (filled bars). The total inhaled doses for humans and mice are 180 and 1.2 µg, respectively.


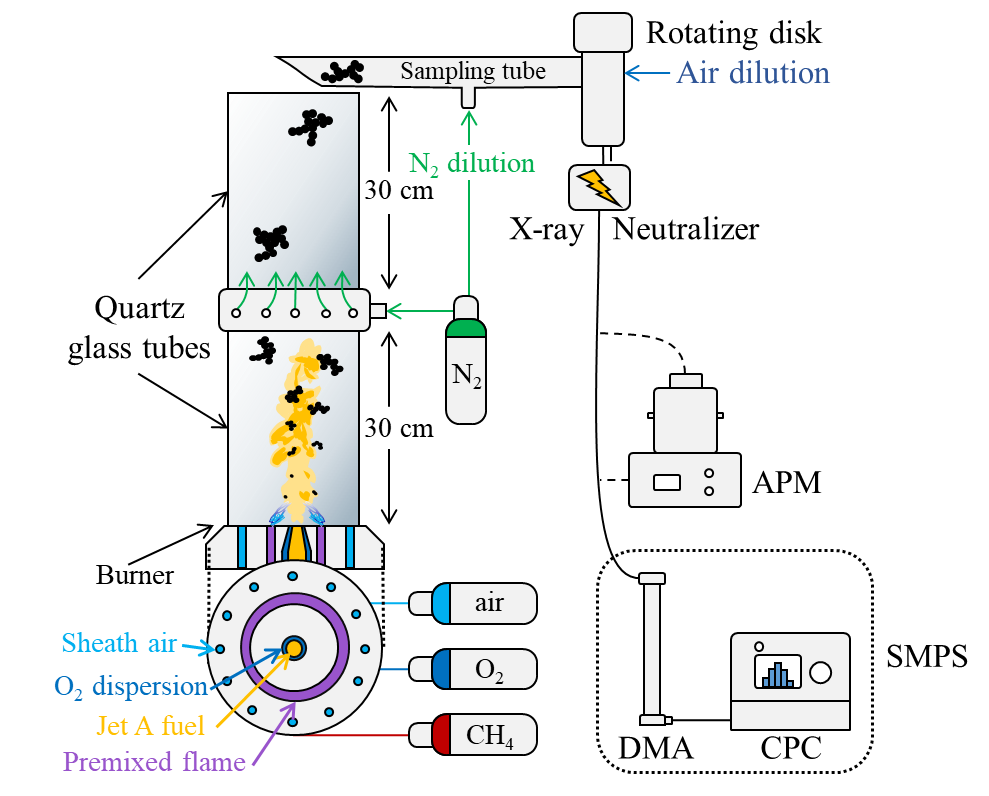


**Figure S3**. Schematic of the experimental set up for preparation of aircraft-like black carbon (BC) nanoparticles from enclosed spray combustion of jet fuel [78]. Jet A fuel is atomized and combusted using an external-mixing twin fluid nozzle enclosed in two, 30 cm long quartz tubes in series. The BC nanoparticles emitted by the reactor are sampled using a straight tube, diluted with N_2_ and air using a rotating disk dilution system, and directed through an X-ray neutralizer to a scanning mobility particle sizer (SMPS) made of a differential mobility analyzer (DMA) coupled with a condensation particle counter (CPC).The SMPS is interfaced with an aerosol particle mass (APM) analyzer to measure the particle mass.


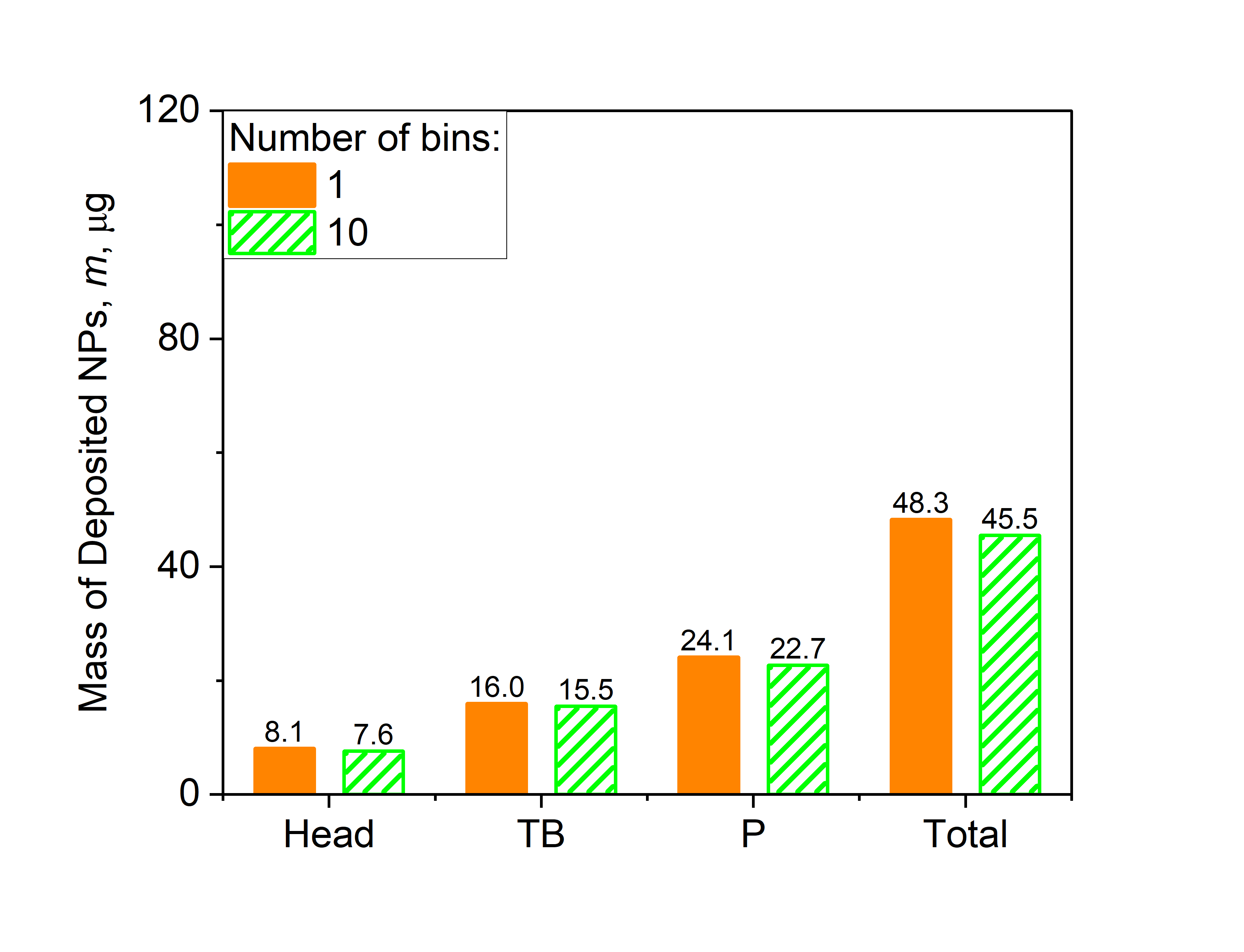


**Figure S4**. Mass of deposited nanoparticles of aircraft BC in human respiratory tract derived by MPPD for a 40-hour exposure based on one bin (filled bars; MMAD = 83.2 nm, ρ_eff_ = 0.34 g/cm^3^) or the mass-weighted average of 10 bins (lined bars) used to discretize the aircraft BC d_m_ distribution (Table S3). The total inhaled dose is 180 µg.

**Table S1.** Summary of the mass-mobility prefactor, *k*, and exponent, *D_fm_*, derived by fitting Eq. 2 to the measured *ρ_eff_* shown in Figure 1.

|  | *k*, g/(nm*^Dfm^*) | *D_fm_* |
| --- | --- | --- |
| Aircraft BC | 1.78·10^-21^ | 2.56 |
| Diesel BC | 5.64·10^-21^ | 2.38 |
| Wood Smoke | 5.65·10^-21^ | 2.38 |
| Silica | 1.46·10^-20^ | 2.10 |
| Zirconia | 2.39·10^-20^ | 2.14 |

**Table S2.** Summary of parameters used for the particle deposition calculations for humans and mice using MPPD model (V3.04).

| Species | Human | | Mouse |
| --- | --- | --- | --- |
| Model | Yeh / Schum Symmetric | | BALB/c |
| Functional Residual Capacity | 3300 mL | | 0.3 mL |
| URT Volume | 50 mL | | 0.0322 mL |
| Exposure | Constant | | |
| Body Orientation | Upright | On Stomach | |
| Aerosol Concentration | 0.01 mg/m^3^ (EPA, January 2023) [100] | | |
| Breathing Frequency | 12 breaths/minute | | 224 breaths/minute |
| Tidal Volume | 625 mL | | 0.22 mL |
| Inspiratory Fraction | 0.5 | | |
| Breathing Scenario | Nasal | | Whole Body Exposure |
| Deposition and Clearance | Deposition Only | | |

**Table S3**. Mobility diameter, d_m_, Count Median Diameter, CMD, Mass Median Aerodynamic Diameter, MMAD, bin median effective density, ρ_eff_, and mass fraction, χ, of aircraft BC discretized into 10 d_m_ bins. MMAD and ρ_eff_ values were used for input into MPPD calculations of lung deposition in humans (Figure S4, 40-hour exposure, total inhaled dose 180 µg).

| Bin # | *d_m_*, nm | CMD, nm | MMAD, nm | *ρ_eff_*, g/cm^3^ | *χ* |
| --- | --- | --- | --- | --- | --- |
| 1 | 0-30 | 15 | 15.4 | 1.0295 | 0.0032 |
| 2 | 30-60 | 45 | 29.6 | 0.6338 | 0.0158 |
| 3 | 60-90 | 75 | 41.5 | 0.5058 | 0.0335 |
| 4 | 90-120 | 105 | 53.0 | 0.4360 | 0.0572 |
| 5 | 120-150 | 135 | 64.4 | 0.3902 | 0.0810 |
| 6 | 150-180 | 165 | 75.6 | 0.3571 | 0.1115 |
| 7 | 180-210 | 195 | 87.0 | 0.3317 | 0.1767 |
| 8 | 210-240 | 225 | 98.3 | 0.3114 | 0.1523 |
| 9 | 240-270 | 255 | 109.7 | 0.2946 | 0.2809 |
| 10 | 270-300 | 278.8 | 118.6 | 0.2832 | 0.0879 |
